# Differential effects of fish-oil and cocoa-butter based high-fat/high-sucrose diets on endocrine pancreas morphology and function in mice

**DOI:** 10.3389/fendo.2024.1265799

**Published:** 2024-02-13

**Authors:** Shaima Albeloushi, Amal Hasan, Hossein Arefanian, Sardar Sindhu, Fatema Al-Rashed, Shihab Kochumon, Nermeen Abukhalaf, Texy Jacob, Steve Shenouda, Ashraf Al Madhoun, Fahd Al-Mulla, Rasheed Ahmad

**Affiliations:** ^1^ Immunology and Microbiology Department, Dasman Diabetes Institute, Dasman, Kuwait; ^2^ Translational Research Department, Dasman Diabetes Institute, Dasman, Kuwait; ^3^ Animal and Imaging Core Facility, Dasman Diabetes Institute, Dasman, Kuwait

**Keywords:** fish oil, cocoa butter, high-fat/sucrose diet, insulin, glucagon, α-cell, β-cell

## Abstract

**Introduction:**

A high-fat/high-sucrose diet leads to adverse metabolic changes that affect insulin sensitivity, function, and secretion. The source of fat in the diet might inhibit or increase this adverse effect. Fish oil and cocoa butter are a significant part of our diets. Yet comparisons of these commonly used fat sources with high sucrose on pancreas morphology and function are not made. This study investigated the comparative effects of a fish oil-based high-fat/high-sucrose diet (Fish-HFDS) versus a cocoa butter-based high-fat/high-sucrose diet (Cocoa-HFDS) on endocrine pancreas morphology and function in mice.

**Methods:**

C57BL/6 male mice (n=12) were randomly assigned to dietary intervention either Fish-HFDS (n=6) or Cocoa-HFDS (n=6) for 22 weeks. Intraperitoneal glucose and insulin tolerance tests (IP-GTT and IP-ITT) were performed after 20-21 weeks of dietary intervention. Plasma concentrations of c-peptide, insulin, glucagon, GLP-1, and leptin were measured by Milliplex kit. Pancreatic tissues were collected for immunohistochemistry to measure islet number and composition. Tissues were multi-labelled with antibodies against insulin and glucagon, also including expression on Pdx1-positive cells.

**Results and discussion:**

Fish-HFDS-fed mice showed significantly reduced food intake and body weight gain compared to Cocoa-HFDS-fed mice. Fish-HFDS group had lower fasting blood glucose concentration and area under the curve (AUC) for both GTT and ITT. Plasma c-peptide, insulin, glucagon, and GLP-1 concentrations were increased in the Fish-HFDS group. Interestingly, mice fed the Fish-HFDS diet displayed higher plasma leptin concentration. Histochemical analysis revealed a significant increase in endocrine pancreas β-cells and islet numbers in mice fed Fish-HFDS compared to the Cocoa-HFDS group. Taken together, these findings suggest that in a high-fat/high-sucrose dietary setting, the source of the fat, especially fish oil, can ameliorate the effect of sucrose on glucose homeostasis and endocrine pancreas morphology and function.

## Graphical Abstract

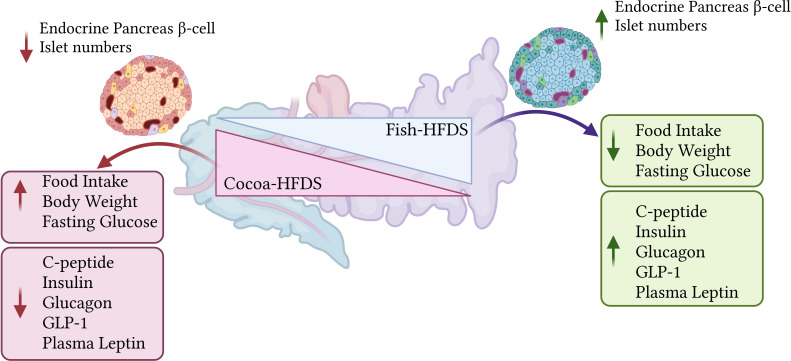

## 1 Introduction

The macronutrients in the diet play an important role in developing metabolic syndrome (1). It has been found that excessive consumption of fat and sugar (a high-energetic diet) is a risk factor for metabolic diseases, and the quality and quantity of ingested dietary components are directly correlated with obesity and diabetes (2, 3). High fat and sugar diet impairs peripheral glucose transport, which is associated with obesity and insulin resistance (4). Type 2 diabetes is commonly associated with impairments in insulin sensitivity and secretion. Macronutrient composition of the diet determines the quality of insulin action (5, 6).

*In vivo*, dietary factors such as high-fat or high-sucrose diets impair insulin action (1, 5–7). Using a model of insulin resistance (8, 9), studies have shown that a high-fat/high-sucrose diet is associated with impaired glucose-stimulated insulin secretion (GSIS) that might affect pancreatic β-cells (10). Long-term exposure to high quantities of fat can lead to β-cell dysfunction (8). Indeed, a high-fat diet has been shown to reduce GSIS despite an absolute increase in the islet insulin content (3). Moreover, diets rich in saturated fatty acids play a role in obesity and insulin resistance (11). However, the type of dietary fat affects the endocrine pancreas function and morphology (12), as well as insulin secretion and sensitivity (7), and is associated with obesity and metabolic disease (11). Similarly, animals fed a high sucrose diet show deleterious effects on glucose homeostasis. Rats fed a high sucrose diet for an extended period developed hyperglycemia and insulin resistance (13, 14), while mice fed high sucrose diet developed glucose intolerance and impaired insulin secretion (9).

Various studies have emphasized the beneficial effects of dietary fish oil and its protective role against metabolic diseases. Dietary fish oil can improve or reverse dyslipidemia, adiposity, β-cell dysfunction, insulin secretion, and insulin action on glucose metabolism (1, 8, 14, 15). A high-fish oil diet has been shown to attenuate obesity and related systemic effects such as insulin resistance (11). In addition, supplementation of a high-fat/high-sucrose diet with fish oil restores glucose homeostasis and insulin sensitivity without changing plasma insulin concentration (1, 14, 16). Furthermore, fish oil supplementation can improve islet and β-cell hypertrophy with the amelioration of insulin resistance (17).

In the present study, we aimed to compare the effects of distinct dietary source of high fat (fish oil versus cocoa butter) in the context of a high-fat/high-sucrose diet on glucose tolerance, insulin resistance and secretion, and endocrine pancreas morphology in mice.

## 2 Research design and methods

### 2.1 Experimental design

The study was approved by the Animal Care and Ethics Committee of the Dasman Diabetes Institute and registered under the identifier RA AM 2016-007. All experiments were conducted in accordance with the National Institutes of Health guidelines for the care and use of laboratory animals.

C57BL/6 mice were purchased from the Jackson Laboratory (Bar Harbor, Maine, USA) and housed in a temperature-controlled environment (23°C) with a relative humidity of 50–60% under 12 h/12 h light/dark cycles. Mice were provided with a standard chow diet and water *ad libitum*.

At 8–10 weeks of age, 12 male mice were randomly assigned to one of two calorie-equivalent diets containing high sucrose and 45% fat as either fish oil-based high-fat/high-sucrose diet (Fish-HFDS; D21042007i, Research Diets Inc, USA) or cocoa butter-based high-fat/high-sucrose diet (Cocoa-HFDS; D21042206i, Research Diets Inc, USA) (**Table 1**) (n = 6/group) and the animals were fed on these diets for 22 weeks. The body weights were measured prior to the diet intervention and weekly during the intervention, along with their food intake.

**Table 1:** Nutritional composition of HFDs.

	Cocoa-HFDS	Fish-HFDS	Chow	HFD
Components %				
Protein	23.7	23.7	14.3	23.7
Carbohydrate	41.4	41.4	6.0	41.4
Fat	23.6	23.6	5.9	23.6
Kcal/gm	4.73	4.73	3.86	4.73
Ingredient %				
L-Cystine	3.0	3.0	0.21	3.0
Corn Starch	20.6	20.6	47.7	195.6
Sucrose	175	175	1.07	0
Cocoa Butter	202.5	0	0	0
Menhaden Oil	0	202.5	0	0
Palm Oil	0	0	0	202.5
Minerals	10	10	10	10
Vitamin Mix	10	10	10	10

### 2.2 Intraperitoneal glucose tolerance test

At 20 weeks of dietary intervention, mice were fasted overnight (12 hours) with free access to water. Fasting blood glucose concentration was measured at 0 minutes, and then, a bolus infusion of glucose (1 g/kg body weight) was administered IP to each mouse. Blood glucose concentrations were measured at 10, 20, 30, 60, 90, and 120 minutes post-glucose infusion using a portable glucometer (Accu-chek, Roche Diagnostic, Germany).

### 2.3 Insulin tolerance test

At 21 weeks of dietary intervention, mice were fasted for 4 hours, and a bolus infusion of insulin (0.8 U/kg body weight) was administered IP. Blood glucose concentrations were measured at 0, 10, 20, 30, 60, 90, and 120 minutes post-insulin infusion using a portable glucometer.

### 2.4 Organ/tissue collection

After 22 weeks of dietary intervention, the mice were euthanized. Organs were identified, removed, weighed, and selected tissues were preserved. The pancreas were fixed in 10% neutral buffered formalin for 24 hours and then stored in 70% ethanol at 4°C until processing. Blood samples were collected from the heart, and plasma was separated and stored at -80°C for further analysis.

### 2.5 Plasma metabolic markers

The concentrations of plasma metabolic markers were measured using a Milliplex kit (MILLIPLEX _MAP_ mouse metabolic magnetic bead panel kit, Millipore, USA), in accordance with the manufacturer’s instructions.

### 2.6 Immunohistochemistry

The fixed pancreas was paraffin-embedded, and tissues were microsectioned by microtomy and labelled with multiple immunofluorescent antibodies, as previously described (18). Briefly, tissue samples on slides were deparaffinized and rehydrated, and then antigen retrieval was carried out. Samples were blocked with 10% goat serum and the following primary antibodies were used for each slide: guinea pig anti-insulin, rabbit anti-glucagon, rabbit anti-insulin and guinea pig anti-pdx1 antibodies. Secondary antibodies used were goat anti-guinea pig Alexa Flour 488 and goat anti-rabbit Alexa flour 546 antibodies (**Table 2**). The antibodies were visualized using an Olympus BX53 upright microscope connected to an Olympus DP73 camera (Olympus, Tokyo, Japan) and an inverted Zeiss LSM710 spectral confocal microscope (Carl Zeiss, Gottingen, Germany). Insulin- and glucagon-positive areas were digitally quantified using ImageJ (Fiji is just ImageJ version 2.9.0/1.53t, Maryland, USA).

**Table 2:** Primary and secondary antibodies used insulin and glucagon staining.

Target/Protein	Host	Clonality	Isotype	Dilution	Supplier	Catalogue #
Insulin	Guinea pig	Polyclonal	IgG	1:150	ThermoFisher	PA1-26938
Glucagon	Rabbit	Polyclonal	IgG	1:150	Cell Signaling	2760
Insulin	Rabbit	Polyclonal	IgG	1:300	Cell Signaling	4590
PDX1	Guinea pig	Polyclonal	IgG	1:300	Abcam	AB47308
Anti-guinea pig Alex Flour 488	Goat	Polyclonal	IgG (H+L)	1:500	ThermoFisher	A11073
Anti-rabbit Alex Flour 546	Goat	Polyclonal	IgG (H+L)	1:500	ThermoFisher	A10040

### 2.7 Endocrine cell mass calculation

β-cells and α-cells were marked and calculated as areas positive for insulin and glucagon staining, respectively. The β-cell area is an area of both positive insulin area and β-cell numbers.


$$\text{β-cell mass}\left(\text{mg}\right)=\frac{\text{β-cell area per field of view}\left({\text{μm}}^{2}\right)\times \text{pancreas weight}\left(\text{mg}\right)}{\text{Tissue area per field of view}\left({\text{μm}}^{2}\right)}$$

The α-cell mass was measured from the above equation (18), using glucagon positive area and α-cell numbers. The total islet cell area was calculated as a sum of the β-cell and α-cell areas per field of view.

### 2.8 Real-time quantitative RT-PCR

Total RNA was extracted from pancreatic tissues using RNeasy Mini Kit (Qiagen, Valencia, CA, USA) as per the manufacturer’s instructions. The cDNA was synthesized using 1 μg of total RNA using a high-capacity cDNA reverse transcription kit (Applied Biosystems, Foster City, CA, USA). Real-time PCR was performed on a QuantStudio™ 5 Real-Time PCR System (Applied Biosystems, Foster City, CA, USA) using an RT^2^ SYBR Green qPCR Mastermix (Qiagen, USA). Each reaction contained 50 ng cDNA that was amplified with primers specific to genes including *Pdx1*, *Ins1, Mafa, Gcg, Mafb, and Gapdh* (**Table 3**), selected from PrimerBank and primer blast website. The threshold cycle (Ct) values of the *Pdx1* gene expression were normalized to the expression of housekeeping gene Gapdh and the expression of other endocrine gene markers was normalized to the expression of *Pdx1* gene. The amounts of target mRNA relative to the control were calculated using the -2ΔΔCt-method. Relative mRNA expression was shown as a fold expression level over that of control expression.

**Table 3:** List of primers.

Target/Protein	
mPdx1-F1	CCCCAGTTTACAAGCTCGCT
mPdx1-R1	CTCGGTTCCATTCGGGAAAGG
mIns1-F1	CACTTCCTACCCCTGCTGG
mIns1-R1	ACCACAAAGATGCTGTTTGACA
mGCG-F1	TTACTTTGTGGCTGGATTGCTT
mGCG-R1	AGTGGCGTTTGTCTTCATTCA
mMAFA-F1	AGGAGGAGGTCATCCGACTG
mMAFA-R1	CTTCTCGCTCTCCAGAATGTG
mMAFB-F1	TTCGACCTTCTCAAGTTCGACG
mMAFB-R1	TCGAGATGGGTCTTCGGTTCA
mGAPDH-F1	TCGGTGTGAACGGATTTG
mGAPDH-R1	GGTCTCGCTCCTGGAAGA
mBactin-F	AAATCGTGCGTGACATCAAA
mBactin-R	AAGGAAGGCTGGAAAAGAGC
mS18-f	CAGCTCCAAGCGTTCCTGG
mS18-r	GGCCTTCAATTACAGTCGTCTTC

### 2.9 Statistical analysis

Statistical analysis was conducted using JMP (version 17.0.0, SAS Institute Inc., North Carolina, USA) and GraphPad Prism (version 9.5.0, GraphPad Software, Inc., California, USA). Data were analyzed by two-way analysis of variance (ANOVA). Significant differences identified by ANOVA were followed by *post hoc* analysis student t-test. The area under the curve (AUC) for IP-GTT and ITT was calculated using the trapezoid rule. Pearson’s correlation was used to examine bivariate relationships, and the correlation coefficient (r) was calculated. *P* ≤ 0.05 was considered statistically significant. Results are reported as mean ± SEM values.

## 3 Results

### 3.1 Effect of fish-HFDS and cocoa-HFDS on food intake and weight gain

**Figure 1:**
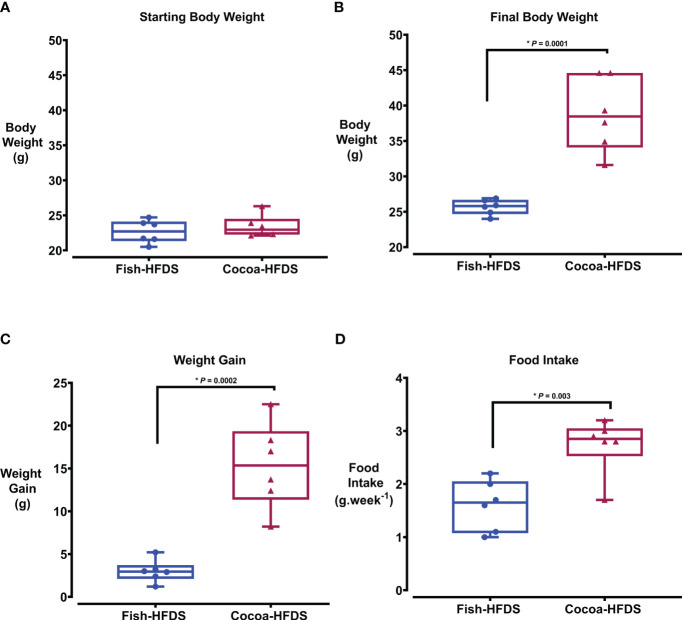
The Effect of different fat-based diets (Fish-HFDS and Cocoa-HFDS) on weight gain and food intake in mice. **(A)** The starting body weight of both groups. **(B)** The final body weight of both groups. **(C)** Weight gain after the diet intervention. **(D)** Food intake during the diet intervention. Data are mean with ± SEM. P-values for differences between groups by ANOVA and Student’s t-test.

Bodyweight and food intake were monitored in both groups throughout the diet intervention. There was no significant difference in the starting body weight between Fish-HFDS and Cocoa-HFDS group (**Figure 1A**). However, after the dietary intervention, body weight of Cocoa-HFDS group was significantly (*P* = 0.0001) higher than that of Fish-HFDS group (**Figure 1B**). In fact, the Cocoa-HFDS group gained five times more weight than the Fish-HFDS group (**Figure 1C**). The Fish-HFDS group had significantly (*P* = 0.003) lower food intake compared to Cocoa-HFDS, which was consistent with the steady low increase in body weight (**Figure 1D**).

### 3.2 Effect of fish-HFDS and cocoa-HFDS on glucose homeostasis and islet function

**Figure 2:**
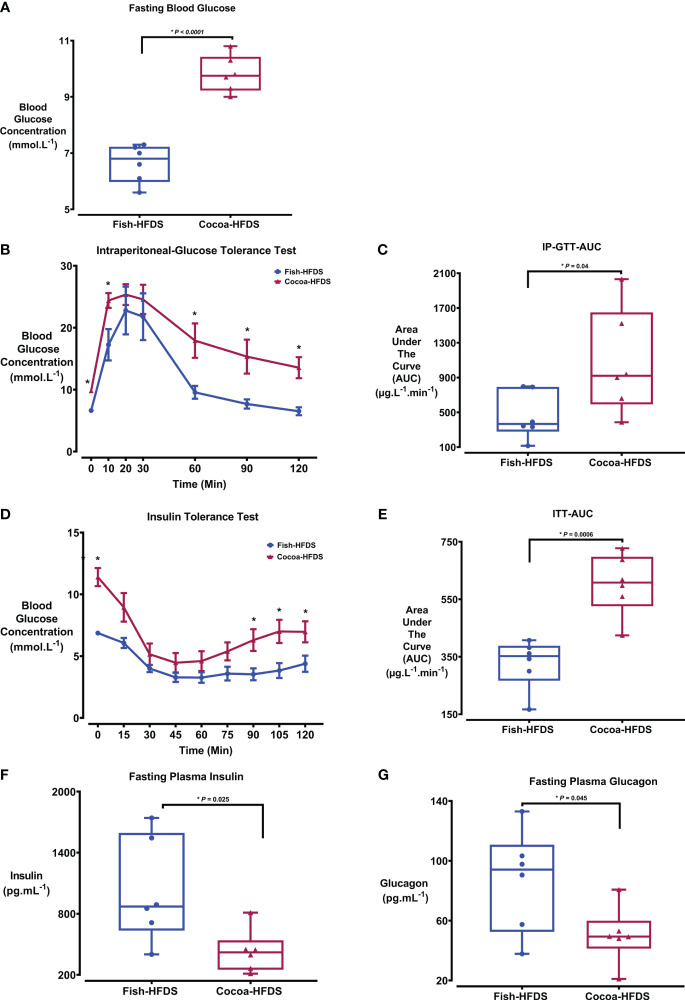
The Effect of different fat-based diets (Fish-HFDS and Cocoa-HFDS) on glucose insulin and glucagon. **(A)** Fasting blood glucose concentration of both groups. **(B)** Intraperitoneal glucose tolerance test (IPGTT). **(C)** AUC _IP-GTT_. **(D)** Insulin tolerance test (ITT). **(E)** AUC _ITT_. **(F)** Fasting plasma insulin concentration. **(G)** Fasting plasma glucagon concentration. Data are mean with ± SEM. *P*-values for differences between groups by ANOVA and Student’s t-test. The asterisk (*) represent a P-value of ≤ 0.05.

To investigate the effect of Fish-HFDS and Cocoa-HFDS on glucose homeostasis, IP-GTT and ITT were performed. The fasting blood glucose concentration was significantly (*P* < 0.0001) less in Fish-HFDS group (6.6 ± 0.3 mmol.L^-1^) compared to Cocoa-HFDS group (**Figure 2A**; **Table 4**). IP-GTT showed that clearance of plasma glucose concentration following intraperitoneal injection of glucose was significantly (*P* = 0.04) higher in mice fed Fish-HFDS compared to mice fed Cocoa-HFDS (**Figure 2B**). Furthermore, the improved glucose tolerance was reflected in the reduction of the AUC _IP-GTT_ (462 ± 112 µg.L^-1^.min^-1^) (**Figure 2C**). Regarding ITT, the mice fed Fish-HFDS showed a greater reduction in blood glucose concentrations after intraperitoneal injection of insulin compared to mice fed Cocoa-HFDS (**Figure 2D**). Moreover, the increased insulin sensitivity in mice fed Fish-HFDS was displayed by the reduction of the AUC _ITT_ (327 ± 35 µg.L^-1^.min^-1^, *P* = 0.0006) compared to mice fed Cocoa-HFDS (**Figure 2E**).

**Table 4:** The Effect of different fat-based diets (Fish-HFDS and Cocoa-HFDS) on plasma fasting blood glucose, insulin, and glucagon.

	Group				Significance (*P-*value)
	Cocoa-HFDS	Fish-HFDS	Chow	HFD	
	N=6	N=6	N=6	N=6	
	Mean	Mean	Mean	Mean	
F.B Glucose	11.5 ± 0.7 ^b^	6.5 ± 0.2 ^a^	5.9 ± 0.3 ^a^	10 ± 0.6 ^b^	**< 0.0001**
Insulin	429 ± 86 ^b^	1023 ± 209 ^a^	2070 ± 216 ^a^	537 ± 103 ^b^	**0.02**
Glucagon	50 ± 8 ^b^	87 ± 14^a^	83.5 ± 9 ^a^	57 ± 14 ^b^	**0.04**

Bold font indicates significance on ANOVA. Non-matching letters indicate a significant difference (P < 0.05) amongst groups on post hoc analysis. Cocoa-HFDS, High-fat-high-sucrose diet with Cocoa butter; Fish-HFDS, High-fat-high-sucrose diet with Fish oil; HFD, normal high-fat diet; F.B Glucose, fasting blood glucose.

Next, we measured the fasting blood insulin in both groups to assess whether Fish-HFDS and Cocoa-HFDS had distinct effects on blood insulin. There was a significant difference in fasting plasma insulin concentrations between the Fish-HFDS and Cocoa-HFDS groups, with mice fed Fish-HFDS having higher concentrations (1023 ± 209 pg.mL^-1^, *P* = 0.02) (**Figure 2F**; **Table 4**).

Glucagon is a peptide hormone secreted from α-cells of the pancreatic islets, and its secretion is linked to plasma insulin levels. Therefore, we wanted to see whether these different high-fat/high-sucrose diets had distinct effects also on blood glucagon concentrations. Our results showed that mice fed Fish-HFDS displayed high fasting blood glucagon concentrations compared to mice fed Cocoa-HFDS (87 ± 14 pg.mL^-1^, *P* = 0.04) (**Figure 2G**; **Table 4**).

### 3.3 Effect of fish-HFDS and cocoa-HFDS on plasma metabolic markers

**Figure 3:**
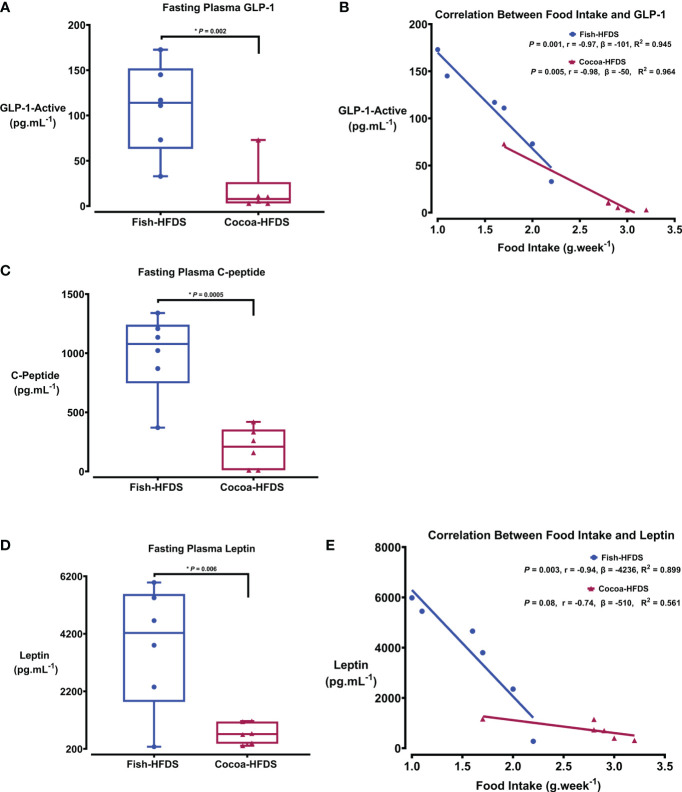
The Effect of different fat-based diets (Fish-HFDS and Cocoa-HFDS) on plasma metabolic markers. **(A)** Fasting plasma GLP-1concentration. **(B)** Correlation between food intake and GLP-1. **(C)** Fasting plasma C-peptide concentration. **(D)** Fasting plasma leptin concentration. **(E)** Correlation between food intake and leptin. Data are mean with ± SEM. *P*-values for differences between groups by ANOVA and Student’s t-test.

Metabolic hormones are affected by high-fat/high-sucrose diets and obesity. We wanted to see whether Fish-HFDS and Cocoa-HFDS had distinct effects on the metabolic markers. Our results showed that glucagon-like peptide (GLP-1) concentrations were significantly (108 ± 20 pg.mL^-1^, *P* = 0.002) higher in Fish-HFDS group compared to Cocoa-HFDS group (**Figure 3A**). A negative correlation was observed between food intake and GLP-1 in both groups (Fish-HFDS group: r = -0.97, *P* = 0.001; Cocoa-HFDS group: r = -0.98, *P* = 0.005) (**Figure 3B**). The Fish-HFDS group had significantly (991 ± 14 pg.mL^-1^, *P* = 0.0005) higher C-peptide concentrations than the Cocoa-HFDS group (**Figure 3C**). Plasma leptin concentrations were significantly (3752 ± 871 pg.mL^-1^, *P* = 0.006) higher in Fish-HFDS group compared to Cocoa-HFDS group (**Figure 3D**). An inverse correlation was found between food intake and leptin levels in the Fish-HFDS group only (r = -0.94, *P* = 0.003) (**Figure 3E**). Other metabolic markers such as secretin, peptide YY, interleukin (IL)-6, pancreatic polypeptide, glucose-dependent insulinotropic polypeptide-total, and tumor necrosis factor (TNF-α) were also measured, but no significant differences were found between the two dietary groups (**Table 5**).

**Table 5:** The Effect of different fat-based diets (Fish-HFDS and Cocoa-HFDS) on plasma metabolic markers.

Plasma Metabolic Markers (pg.mL^-1^)	Group		Significance (*P-*value)
	Cocoa-HFDS	Fish-HFDS	
	N=6	N=6	
	Mean	Mean	
Secretin	94 ± 21	61 ± 17	0.25
Ghrelin-Active	130 ± 24	41 ± 13	**0.008**
Peptide YY	191 ± 25	232 ± 22	0.24
Interleukin-6	32 ± 5	32 ± 6	0.98
GLP-1	17 ± 11	108 ± 20	**0.002**
C-peptide	198 ± 69	991 ± 140	**0.0005**
Leptin	741 ± 146	3752 ± 871	**0.006**
Pancreatic Polypeptide	118 ± 35	119 ± 34	0.98
GIP	144 ± 31	241 ± 50	0.13
TNF-α	12 ± 2	14 ± 4	0.69

Bold font indicates significance in Student’s t-test. Cocoa-HFDS, High-fat-high-sucrose diet with Cocoa butter; Fish-HFDS, High-fat-high-sucrose diet with Fish oil; GLP-1, Glucagon-Like Peptide-1-Active; GIP, Glucose-dependent Insulinotropic Polypeptide-Total; TNF-α, Tumour Necrosis Factor.

### 3.4 Effect of fish-HFDS and cocoa-HFDS on endocrine pancreas morphology and integrity

**Figure 4:**
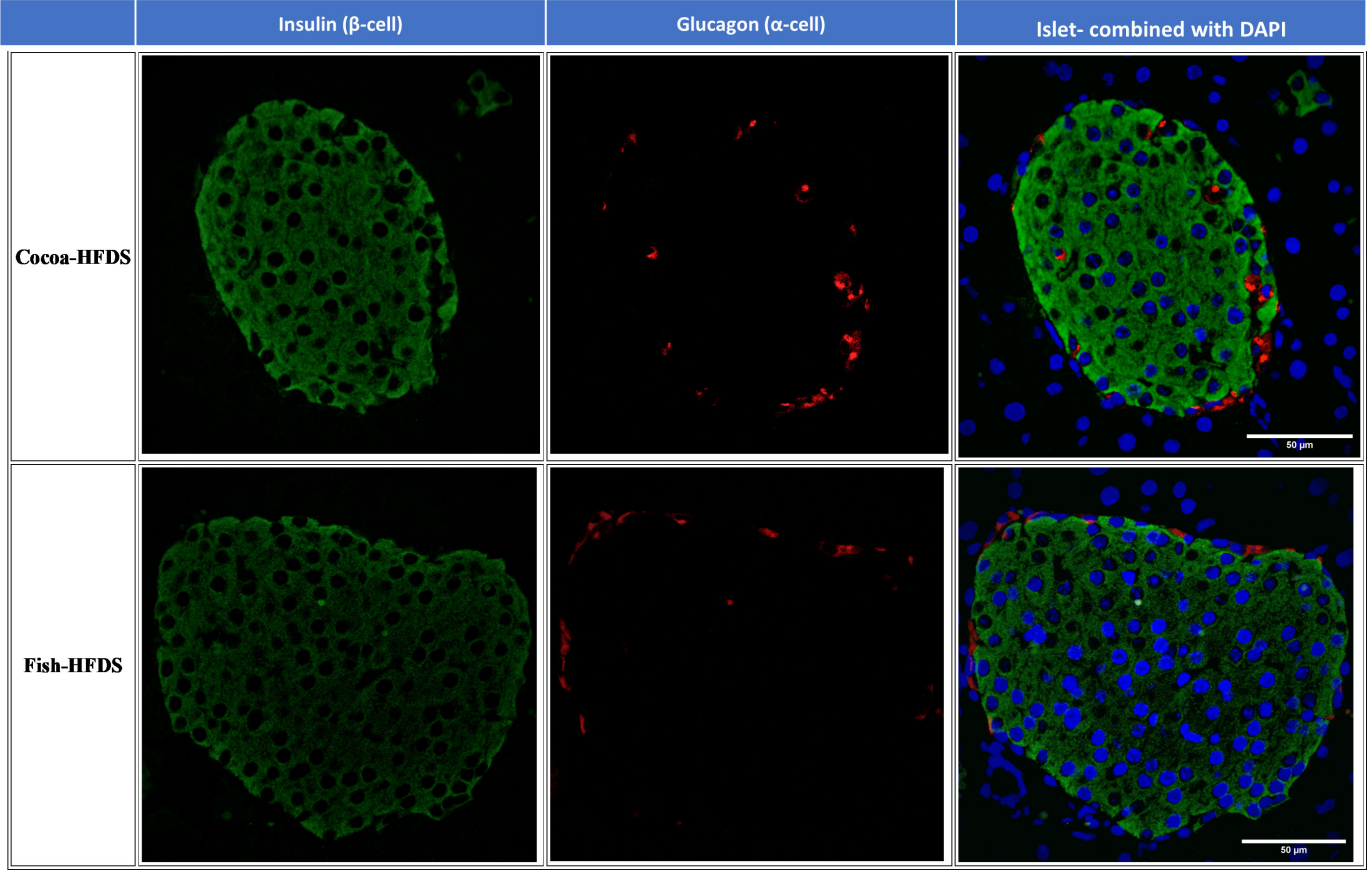
Representative photomicrographs of double staining of insulin and glucagon. Scale bar 50 µm.

**Figure 5:**
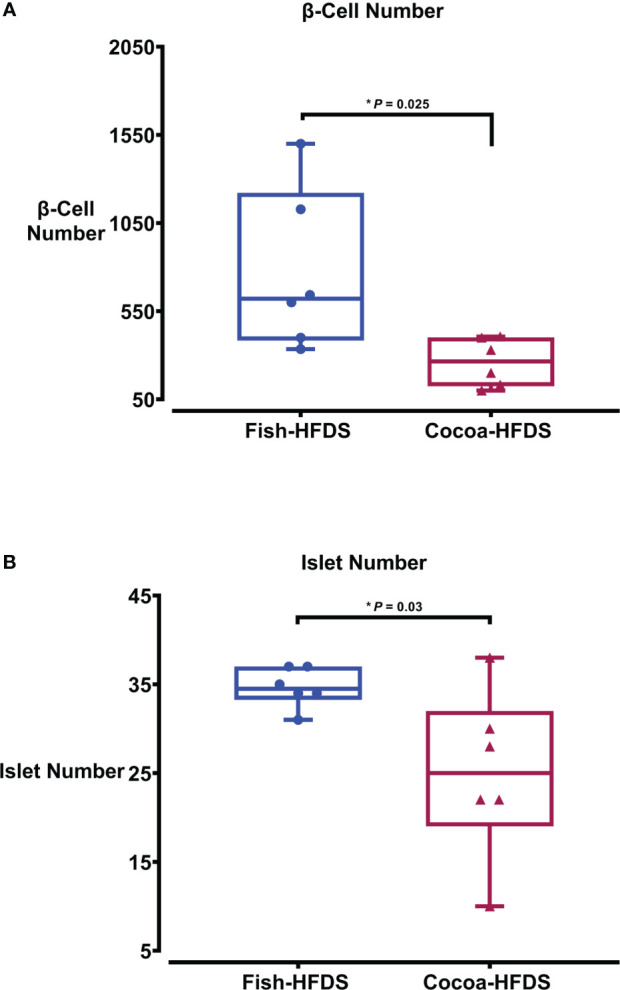
The Effect of different fat-based diets (Fish-HFDS and Cocoa-HFDS) on endocrine pancreas morphology. **(A)** Number of β-cells. **(B)** Number of islets. Data are mean with ± SEM. *P*-values for differences between groups by ANOVA and Student’s t-test.

**Figure 6:**
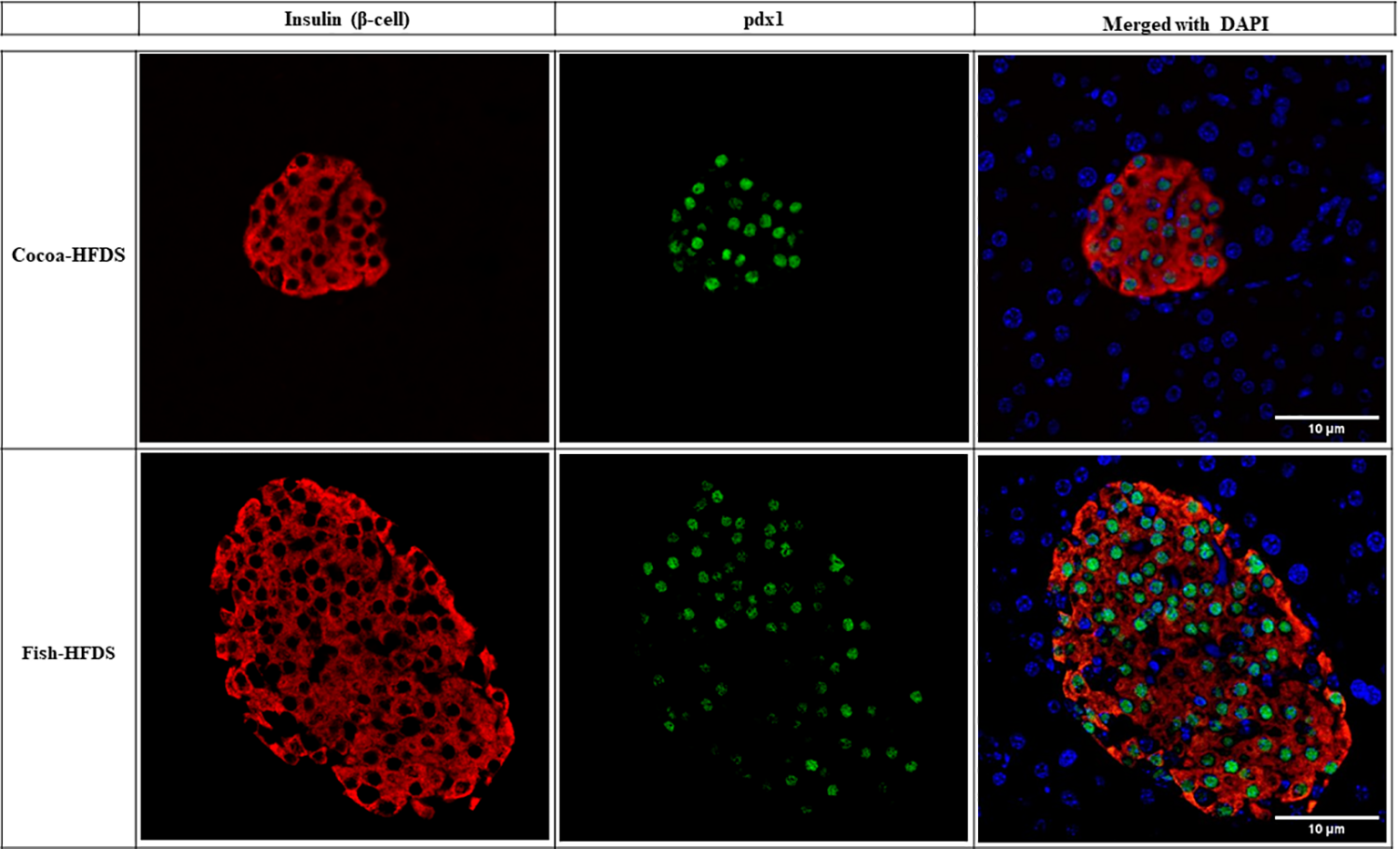
Representative photomicrographs of double staining of insulin and *pdx1*. Scale bar 10 µm.

When pancreas sections were double stained for insulin and glucagon, the endocrine sections of the pancreas represented by the islets of Langerhans were distributed within the exocrine section of the pancreas (**Figure 4**). Our pancreatic histology data showed that α-cells, β-cells, and islet numbers were higher in Fish-HFDS group compared to Cocoa-HFDS group. However, only β-cells and islet numbers reached statistical significance (*P* = 0.025, *P* = 0.03, respectively) (**Figures 5A, B**). Insulin and glucagon-positive areas and α-cells, β-cells, and islet mass were similar between two groups. As a key regulatory marker of endocrine pancreas function, the expression of Pdx1 was detected which differed non-significantly between two groups (**Figure 6**). In addition, we also compared gene expression of *Ins1*, *Gcg* and several critical transcriptional regulators of β-cell (*Pdx1*, *Mafa*) and α cell (*Pdx1*, *Mafb)* development. Except the *Ins1* gene expression (*P* = 0.008), all other markers differed non-significantly between two dietary groups (**Table 6**).

**Table 6:** The Effect of different fat-based diets (Fish-HFDS and Cocoa-HFDS) on the gene expression of Insulin, MAFA, Glucagon, MAFB and pdx1.

Genes	Group		Significance (*P-*value)
	Cocoa-HFDS	Fish-HFDS	
	N=6	N=6	
	Mean	Mean	
*Insulin*	0.06 ± 0.02	6.37 ± 1.98	**0.008**
*MAFA*	1.18 ± 0.51	1.07 ± 0.17	0.81
*Glucagon*	0.02 ± 0.01	15.92 ± 9.05	0.052
*MAFB*	0.75 ± 0.03	1.07 ± 0.17	0.13
*pdx1*	1.05 ± 0.09	1.01 ± 0.05	0.71

Bold font indicates significance in Student’s t-test. Cocoa-HFDS, High-fat-high-sucrose diet with Cocoa butter; Fish-HFDS, High-fat-high-sucrose diet with Fish oil.

## 4 Discussion

The present study investigated and compared the effects of fish oil and cocoa butter as high-fat/high-sucrose diets on glucose tolerance, β-cell function, insulin sensitivity, and endocrine pancreas morphology and integrity in mice. This study used a mouse model to mimic a human nutritional setting of intake of high-fat diets. The components and ingredients of our diets were similar to the regular HFD, with the difference in the source of fat.

High-sucrose diets are known to increase body weight (2, 6, 10); however, when supplemented with fish oil, using different models, animals either gained (8, 19, 20), lost (21, 22), or showed no change (2, 10, 14, 15, 23–25) in their body weight. Here, we show that fish oil fat source in high fat/high sucrose diet does not, in fact, increase the body weight when compared to a high-fat diet based on cocoa butter as fat source. To determine whether the increase in body weight was due to increased food intake, we assessed the dietary intake of the mice. To this end, the Fish-HFDS group showed a lower food intake compared to Cocoa-HFDS group, which was consistent with the steady low increase in body weight of mice in Fish-HFDS group. Comparing with other studies, mice fed with high-fat/high-sucrose diets supplemented with fish oil either showed an increase (26) or a decrease in the food intake (2). The discrepancies in results of these studies can possibly be attributed to differences with regard to mice ages, fat and sugar percentages in diets, as well as dietary components other than fat and sucrose and feeding durations.

The Cocoa-HFDS group had higher fasting blood glucose concentrations than the Fish-HFDS group, which raises the possibility of a disturbance of glucose metabolism in the Cocoa-HFDS group. It was shown previously by several studies that the presence of fish oil in the high-sucrose diet affects the fasting glucose concentration, albeit within the normal range, in comparison to other groups that had only a high-sucrose diet alone or with a different source of fat (e.g., corn oil, lard, argan oil, and krill oil) (1, 2, 8, 14, 22, 25). In our study, compared to normal (chow) diet and regular HFD, the mice fed with Fish-HFDS had similar fasting blood glucose concentrations as the mice fed with chow diet and had lower fasting blood glucose concentrations than the mice fed with regular HFD.

Insulin resistance is a condition characterized by low peripheral tissue (muscle, liver, and adipose tissues) response to insulin (27, 28). A high-fat/high-sucrose diet is commonly used as a model of diet-induced insulin resistance and impaired glucose homeostasis, shown by the increased fasting glucose concentrations and amplified glucose and insulin responses to IP-GTT (2). Of note, it has been suggested that sucrose *per se* does not affect insulin-glucose uptake (7), in spite of the fact that rats fed a high-sucrose diet showed mild hyperglycemia (8, 16). The best method, considered a gold standard, for determining insulin resistance is the hyperinsulinemic-euglycemic clamp procedure. However, since this technique cannot be implemented routinely, ITT was developed as a simple alternative technique to evaluate insulin action *in vivo* (28). Accordingly, IP-GTT and ITT tests were used in this study to measure and compare the insulin action between Fish-HFDS and Cocoa-HFDS dietary groups. Our data showed that the AUC was lower in Fish-HFDS group compared to Cocoa-HFDS group, indicating a higher blood glucose clearance rate and hence a more proficient glycemic control in mice of Fish-HFDS group.

The Fish-HFDS group showed a better glucose tolerance test outcome, compared to Cocoa-HFDS group indicating that fish oil does not have harmful effects on β-cell sensitivity and may in fact have beneficial effects. This may suggest that the presence of good fat in the diet can either prevent insulin resistance or even promote insulin sensitivity, irrespective of the percentage of fat or calorie intake. The Fish-HFDS group had remarkedly lower fasting blood glucose concentrations compared to Cocoa-HFDS group, which may indicate that the Fish-HFDS group may have had more sensitive β-cells responding to glucose sensing. Moreover, the Fish-HFDS group also had higher blood insulin concentrations compared to Cocoa-HFDS group, which suggests that the fish oil may have had a positive modulatory impact on the β-cell secretory capacity, leading to an increase in insulin secretion. In support of our findings, previous studies have demonstrated that rats fed on high sucrose diet supplemented with fish oil showed normal glucose tolerance tests (1, 2, 16, 23) and did not develop insulin resistance. This indicates that the fish oil ameliorated and prevented insulin resistance (5). Of note, although previous studies have suggested that a high-fat diet impairs glucose tolerance independent of macronutrient composition (19), our study provides evidence that the type of fat does make a difference.

Moreover, it was suggested that sucrose, but not protein or fat, strongly stimulates pancreatic insulin secretion (19). In our study, the Fish-HFDS group had higher plasma insulin and glucagon concentrations compared to the Cocoa-HFDS group. However, the Fish-HFDS group had lower plasma insulin and glucagon concentrations compared to mice fed normal chow diet and higher plasma insulin and glucagon concentrations than mice fed regular HFD. Therefore, the fish oil as a source of fat in a high-sucrose high-fat diet effectively increased these hormones in mice. However, the previous studies on rats (25) and rabbits (22) fed a high-sucrose diet showed that insulin concentration was either not affected (25) or decreased (22) by fish oil supplementation. We speculate that different feeding regimens used in these dietary investigations, including ours, may be the source of differences in results regarding the circulatory insulin levels. Besides, the diets induce metabolic effects differentially across different species.

Insulin plays a vital role in glucose homeostasis; it inhibits glucose production and stimulates glucose uptake (29). During fasting, glucagon maintains glucose availability by stimulating the hepatic gluconeogenesis. Glucagon secretion is stimulated during hypoglycemia and is suppressed during hyperglycemia (30), providing the first line of defense in glucose counter-regulation (31). However, the balance between glucagon and insulin in the prandial state is responsible for normal glucose tolerance (32). Taken together, glucose homeostasis depends on the cooperation between α- and β-cells, not exclusively on the β-cells (31). Rather than having opposing roles in regulation of glucose homeostasis, α- and β-cells cooperate in the prandial state to regulate the metabolic control of nutrients (33). Besides, the role of certain enzymes such as acetylcholinesterase, α-glycosidase, butyrylcholinesterase, and carbonic anhydrase II could be considered in relation to glucose homeostasis, as some natural products studies have shown potential anti-diabetic effects by targeting these enzymes (34–36). However, glucagon measurements are still rarely reported in animal studies of glucose metabolism, which may represent an area of growing interest in metabolic research studies.

Plasma leptin concentrations were higher in Fish-HFDS group compared to Cocoa-HFDS group. Similar to our findings, previous studies showed that adipose tissue and plasma leptin concentrations were high in rats fed high sucrose, high-fat fish oil based diet (21, 26). In our study, fish oil increased plasma leptin concentrations without significant changes in body weight gains, which is consistent with what was found in previous studies (13, 26). Nonetheless, an inverse correlation was found between food intake and plasma leptin concentrations in the Fish-HFDS group, which is expected considering the metabolic role of leptin as a satiety regulatory hormone.

Our data suggest that high concentrations of GLP-1, and leptin also, could be responsible for the reduced food intake, lower blood glucose concentrations, and less weight gains in the Fish-HFDS group compared to Cocoa-HFDS group, although both groups showed a negative correlation between food intake and GLP-1. Importantly, the Fish-HFDS group had higher C-peptide concentrations than the Cocoa-HFDS group, which indicates a better β-cell function in mice fed Fish-HFDS. However, other studies reported that the C-peptide concentration was not affected by the supplementation of fish oil to a normal diet in both lean and obese rats (37). Clearly, the insulinotropic effect varied as fish oil was added as supplement to a normal diet or fed to mice concurrently as a high-fat high-sucrose diet (the regimen used in our study).

An optimal pancreatic endocrine cell mass is necessary for organ function, and changes in the mass lead to metabolic disorders (12). The pancreatic endocrine cell mass is calculated from the cell size; however, the mass can be increased due to proliferation and hypertrophy or decreased by apoptosis and atrophy (38, 39). Under normal conditions during adulthood, pancreatic β-cells have a steady low rate of proliferation and apoptosis (39). Maintaining β-cell mass during adulthood is vital to maintain blood glucose homeostasis and prevent diabetes (39). Cell mass, especially β-cell mass, can expand during adulthood in response to increased body weight and insulin resistance (38, 39), thereby displaying an ability to adapt to the increased insulin demand (38).

Given the critical role that the endocrine pancreas plays in maintaining blood glucose homeostasis, we assessed endocrine pancreas cell number and expression of endocrine pancreas regulatory markers. Our data showed that the α-cells, β-cells, and islet numbers were higher in Fish-HFDS group compared to Cocoa-HFDS group. However, only β-cells and islet numbers reached statistical significance. The insulin and glucagon-positive areas and α-cells, β-cells, and islet mass were similar between two groups. We observed nonsignificant differences between two dietary groups regarding gene expression of *Pdx1*, *Mafa/b* and *Gcg* while significant difference was observed regarding *Ins*. Other studies showed that when fish oil was added to a normal diet, it did not affect islet size in both obese or lean rats (37). However, a high-fat diet is known to increase pancreatic β-cell mass by both hypertrophy and hyperplasia to produce more insulin as a compensatory mechanism against insulin resistance (10, 40). β-cell hypertrophy and hyperplasia are associated with the loss of β-cell function, leading to a defect in insulin secretion (27). Prolonged insulin resistance leads to the activation of the apoptotic pathways increasing β-cell apoptosis and decreasing β-cell mass (40). β-cell function and β-cell mass are correlated, and both decrease with glucose intolerance (41). Decreased β-cell mass is associated with impaired fasting glucose and glucose tolerance (41); in our study, the Cocoa-HFDS group had high fasting blood glucose concentration and impaired glucose tolerance with no significant difference in β-cell mass compared to the Fish-HFDS group, suggesting an impairment in glucose homeostasis. However, further studies that incorporate additional control groups (high-sucrose or/and normal diet) are needed to confirm these possibilities. Interestingly, many findings suggest that changes in α-cell mass are attributed to glucose intolerance (41). However, we measured α-cell mass and found no difference between two dietary groups. Further research measuring the β-cell apoptosis to proliferation ratios will be needed to confirm whether the increase in β-cell numbers in the Fish-HFDS group is not a compensatory mechanism against the diet-induced insulin resistance. Moreover, pre-intervention with fish oil has a favorable impact on islet morphology (42), which needs to be further evaluated in various settings.

## 5 Conclusion

Our findings suggest that manipulating dietary fats within a high sucrose diet may improve certain features of the metabolic syndrome improving glucose homeostasis. In this regard, replacing the fat content in the high-fat/high-sucrose diet with fish oil seems to be associated with the following: reduced food intake, reduced weight gain, reduced fasting blood glucose concentrations, improved glucose tolerance tests and insulin sensitivity, and a favorable effect on islet morphology. However, the molecular mechanisms that underlie the fish oil effects need to be further investigated.

## Data availability statement

The original contributions presented in the study are included in the article/supplementary material. Further inquiries can be directed to the corresponding author.

## Ethics statement

The animal study and all animal experimental protocols were approved by the Dasman Diabetes Institute Animal Care and Ethics Committee (RA AM- 2016-007). All institutional and national/international guidelines for the care and use of laboratory animals were followed. The study was conducted in accordance with the local legislation and institutional requirements.

## Author contributions

SA: Data curation, Formal Analysis, Investigation, Methodology, Writing – original draft. AH: Formal Analysis, Writing – review & editing. HA: Writing – review & editing, Data curation, Methodology. SaS: Resources, Writing – review & editing. FA-R: Formal Analysis, Writing – review & editing. SK: Data curation, Formal Analysis, Methodology, Writing – review & editing. NA: Data curation, Methodology, Writing – review & editing. TJ: Data curation, Methodology, Writing – review & editing. StS: Data curation, Methodology, Writing – review & editing. AA: Resources, Writing – review & editing. FA-M: Formal Analysis, Resources, Writing – review & editing. RA: Conceptualization, Funding acquisition, Resources, Supervision, Writing – review & editing.
